# Arboviral Infections in Neurological Disorders in Hospitalized Patients in São José do Rio Preto, São Paulo, Brazil

**DOI:** 10.3390/v14071488

**Published:** 2022-07-07

**Authors:** Bruno H. G. A. Milhim, Leonardo C. da Rocha, Ana C. B. Terzian, Carolina C. P. Mazaro, Marcos T. Augusto, Adriana Luchs, Nathalia Zini, Livia Sacchetto, Barbara F. dos Santos, Pedro H. C. Garcia, Rodrigo S. Rocha, Elisabete Liso, Vânia M. S. Brienze, Gislaine C. D. da Silva, Nikos Vasilakis, Cássia F. Estofolete, Maurício L. Nogueira

**Affiliations:** 1 Laboratório de Pesquisas em Virologia [LPV], Faculdade de Medicina de São José do Rio Preto [FAMERP], Avenida Brigadeiro Faria Lima, 5544, Vila São Jose, São José do Rio Preto 15090-000, SP, Brazil; brunohgam@hotmail.com (B.H.G.A.M.); leocecilior@hotmail.com (L.C.d.R.); anacarolinaterzian@gmail.com (A.C.B.T.); carolpacca@gmail.com (C.C.P.M.); marcosaugusto1993@outlook.br (M.T.A.); nathalia_zini@hotmail.com (N.Z.); liviasacchetto@gmail.com (L.S.); barbara_fs13@hotmail.com (B.F.d.S.); pedrohenriquecarrilho1001@gmail.com (P.H.C.G.); rodrigosr12@hotmail.com (R.S.R.); gislaine.cds@gmail.com (G.C.D.d.S.); cassiafestofolete@gmail.com (C.F.E.); 2 Laboratório de Imunologia Celular e Molecular (LICM), Avenida Augusto de Lima, 1715, Centro, Belo Horizonte 30190-002, MG, Brazil; 3 Instituto René Rachou Fundação Oswaldo Cruz, Avenida Augusto de Lima, 1715, Centro, Belo Horizonte 30190-002, MG, Brazil; 4 Enteric Disease Laboratory, Department of Virology, Adolfo Lutz Institute, Avenida Dr. Arnaldo, 355, São Paulo 01246-902, SP, Brazil; driluchs@gmail.com; 5 Hospital de Base, Avenida Brigadeiro Faria Lima, 5544-Vila São Jose, São José do Rio Preto 15090-000, SP, Brazil; eliliso@terra.com.br (E.L.); vania.brienze@hospitaldebase.com.br (V.M.S.B.); 6 Department of Pathology, University of Texas Medical Branch, 301 University Blvd., Galveston, TX 77555-0609, USA; nivasila@utmb.edu; 7 Department of Preventive Medicine and Population Health, The University of Texas Medical Branch, Galveston, TX 77555-1150, USA; 8 Center for Vector-Borne and Zoonotic Diseases, The University of Texas Medical Branch, Galveston, TX 77555-0609, USA; 9 Center for Biodefense and Emerging Infectious Diseases, University of Texas Medical Branch, 301 University Blvd., Galveston, TX 77555-0609, USA; 10 Center for Tropical Diseases, University of Texas Medical Branch, 301 University Blvd., Galveston, TX 77555-0609, USA; 11 Institute for Human Infection and Immunity, University of Texas Medical Branch, 301 University Blvd., Galveston, TX 77555-0610, USA

**Keywords:** arbovirus, neurological disorders, surveillance

## Abstract

Arbovirus infections are increasingly important causes of disease, whose spectrum of neurological manifestations are not fully known. This study sought to retrospectively assess the incidence of arboviruses in cerebrospinal fluid samples of patients with neurological symptoms to inform diagnosis of central and peripheral nervous system disorders. A total of 255 cerebrospinal fluid (CSF) samples collected from January 2016 to December 2017 were tested for dengue virus (DENV 1-4), Zika virus (ZIKV), and Chikungunya virus (CHIKV) in addition to other neurotropic arboviruses of interest, using genetic and serologic assays. Of the 255 CSF samples analyzed, 3.53% (09/255) were positive for arboviruses presenting mainly as meningitis, encephalitis, and cerebrovascular events, of which ZIKV was detected in 2.74% (7/255), DENV in 0.78% (2/255), in addition to an identified ILHV infection that was described previously. All the cases were detected in adults aged 18 to 74 years old. Our findings highlight the scientific and clinical importance of neurological syndromes associated with arboviruses and demonstrate the relevance of specific laboratory methods to achieve accurate diagnoses as well as highlight the true dimension of these diseases to ultimately improve public health planning and medical case management.

## 1. Introduction

Arthropod-borne viruses [arboviruses] are a diverse array of viruses that are the cause of major epidemic worldwide with significant public health consequences [1]. They are maintained in nature by hematophagous arthropod vectors and vertebrate hosts in ecologically and phylogenetically distinct transmission cycles [2]. Arboviruses are members of seven virus families—*Togaviridae*, *Flaviviridae*, *Bunyaviridae*, *Reoviridae*, *Rhabdoviridae*, *Orthomyxoviridae,* and *Asfarviridae*. Arbovirus infections manifest in a broad spectrum of illness ranging from asymptomatic to severe and fatal disease. While most infections result in a non-specific febrile illness that resolves, severe manifestations of disease are often manifest as systemic febrile illness, hemorrhagic fever, and/or invasive neurological disease [3]. For the latter, the most common etiologic agents in South America are members of the *Togaviridae* and *Flaviviridae* families, such as chikungunya (CHIKV), Venezuelan equine encephalitis (VEEV), Madariaga (MADV), Mayaro (MAYV) and dengue (DENV), Zika (ZIKV), Rocio (ROCV), and Ilheus (ILHV) viruses, respectively [4,5,6,7,8,9,10,11].

Although the incidence rate remains uncertain [12], neurological manifestations in DENV infections have been widely reported [13,14,15,16], reviewed in [10], and have ranged from encephalitis [17,18]–the most commonly reported neurological symptom–to seizures [19], mononeuropathy [20,21], cerebrovascular events [14], meningitis [22,23], and Guillain-Barré syndrome (GBS) or Miller-Fisher syndrome [15,24]. The explosive global spread of ZIKV and its introduction in South America was associated with neurological manifestations [25,26] such as congenital Zika syndrome (CZS) [26,27,28,29], neuropathy [30], GBS [31,32], stroke [33], and ocular manifestations [34,35]. Human ILHV infections have been documented throughout Central America, the Caribbean, and South America. Symptoms usually resolve within a week of onset and include fever, headaches, myalgia, muscular weakness, nausea, and photophobia [36]. However, a recent report documented severe manifestations presented as cerebral encephalitis characterized by intraparenchymal hemorrhage in the parietal lobe, surrounding brain edema and intraventricular hemorrhage [9].

The identification of viral etiologic agents causing neurologic disease can be challenging in hyperendemic areas of arbovirus circulation [7]. Currently, a diagnosis of an arboviral neuroinvasive disease is recommended to include an association between clinical and laboratory criteria. Clinical criteria include meningitis, encephalitis, acute flaccid paralysis, or other acute signs of central or peripheral neurologic dysfunction, as documented by the attending physician. Laboratory criteria are based on serum detection of viral RNA or IgM antibodies, and especially in the CSF to establish a diagnosis [37]. Often, public health agencies require diagnoses based on clinical data. However, these viruses have a large overlap of symptoms, which makes it difficult for health care professionals to establish a reliable diagnosis [38,39].

Starting in 2006, an arbovirus surveillance network for encephalitides has been established in the city of São José do Rio Preto (SJdRP), SP, Brazil. The city is located at the northwest corner of the state and is hyperendemic for various arboviruses of significant public health concern, including DENV [38,40,41,42,43,44,45,46,47,48], Saint Louis encephalitis [49,50], ZIKV [38], CHIKV [51], ILHV [9], and others. Herein, we report several cases of neurological disorders in patients hospitalized with arbovirus infections in São José do Rio Preto, SP, Brazil.

## 2. Materials and Methods

### 2.1. Study Design

This study was performed based on samples collected between January 2016 and December 2017 based on cerebrospinal fluid (CSF) samples from hospitalized patients at Hospital de Base de São José do Rio Preto, a tertiary referral center in the prefecture of São José do Rio Preto, SP, Brazil, that includes two million inhabitants and 102 municipalities. The inclusion criterion for the selection of cases was CSF samples collected from suspected CNS infections, whereas exclusion criteria included CNS infections due to bacterial and fungal infections, CNS clinical syndromes without suspected CNS infection, and presence of cranial trauma. CSF samples were stored at −70 °C.

Demographic, clinical, and laboratory data were collected from the electronic records of all patients included in the study. Confidentiality was ensured by de-identifying questionnaires and samples. The criteria adopted to confirm arbovirus infection associated with neurological involvement were CSF with viral infection patterns (e.g., increase in cell counts with a predominance of lymphocytes and monocytes, and elevated CSF protein levels), a PCR that was positive for an arbovirus or arbovirus-specific IgM antibodies detected in the presence of neurological signs, and symptoms such as meningitis, encephalitis, acute flaccid paralysis, or other acute signs of central or peripheral neurologic dysfunction, as documented by a physician.

### 2.2. Molecular Testing

The selected CSF samples were screened for the presence of DENV 1–4, ZIKV, CHIKV, ROCV, SLEV, WNV, MAYV, ILHV, YFV, BSQV, IGUV, and MADV. Viral RNA (vRNA) was extracted from 140 μL of sample using the QIAmp^®^ Viral RNA (QIAGEN, Germantown, MD, USA) following the manufacturer’s recommendations.

The Trioplex real-time reverse transcriptase-polymerase chain reaction (RT-qPCR) assay was performed using the kit provided by the Centers for Disease Control and Prevention specific for Zika, Chikungunya, and all dengue serotypes [52]. A total of 10 µL of vRNA, 0.5 µM of each probe (DENV-P: FAM-CGYCTWTCAATATGC TGAAACGCG-BHQ-1; CHIKV-P: HEX-ACAGTGGTTTCGTGTGAGGGCTAC-BHQ-1; ZIKV-P: TAMRA-AGCCTACCTTGACAAGCAGTCAGACACTCAA-BHQ-1) and primers (DENV-F: TAGTCTRCGTGGACCGACAAG; DENV-R1: CAGTTGACACRCGGTTTCTC; DENV-R2: GGGTTGATACGCGGTTTCTC; CHIKV-F: ACCATCGGTGTTCCATCTAAAG; CHIKV-R: GCCTGGGCTCATCGTTATT; ZIKV-F: CCGCTGCCCAACACAAG; ZIKV-R: CCACTAACGTTCTTTTGCAGACAT), 12.5 µL of the 2X PCR Master Mix, and 0.5 µL of Superscript III RT/Platinum Taq enzyme mix (SuperScript^®^ III Platinum^®^ One-Step qRT-PCR System, Invitrogen, Carlsbad, CA, USA) were applied to a 96-well plate and analyzed in QuantStudio™ Dx instrument (Thermo Fisher Scientific, Waltham, MA, USA) with the following conditions: 50 °C for 30 m, followed by 45 cycles of 95 °C for 15 s, and 60 °C for 1 min. The results were interpreted as being positive when the cycle threshold (Ct) values were less than 36.

One-Step Real time multiplex PCR assays were performed using the GoTaq^®^ Kit (Promega, Madison, WI, USA). In fourplex reaction mixtures, 50 pmol each of DENV-1- and DENV-3-specific primers, 25 pmol each of DENV-2- and DENV-4-specific primers (DENV-1 F: CAAAAGGAAGTCGTGCAATA; DENV-1 C: CTGAGTGAATTCTCTCTACTGAACC; DENV-2 F: CAGGTTATGGCACTGTCACGAT; DENV-2 C: CCATCTGCAGCAACACCATCTC; DENV-3 F: GGACTGGACACACGCACTCA; DENV-3 C: CATGTCTCTACCTTCTCGAC TTGTCT; DENV-4 F: TTGTCCTAATGATGCTGGTCG; DENV-4 C: TCCACCTGAGAC TCCTTCCA), and 9 pmol of each probe (DENV-1 P: FAM-CATGTGGTTGGGAGCACGC-BHQ-1; DENV-2 P: HEX-CTCTCCGAGAACAGGCCTCGACTTCAA-BHQ-1; DENV-3 P- TAMRA-ACCTGGATGTCGGCTGAAGGAGCTTG-BHQ-2; DENV-4 P- Cy5-TTCCTACTCCTACGCATCGCATTCCG-BHQ-3) were combined in a 50-µL volume total reaction mixture. Real-time PCR was performed on a 96-well plate and analyzed in QuantStudio™ Dx instrument (Thermo Fisher Scientific, Waltham, MA, USA) with the following conditions: 50 °C for 30 m, followed by 45 cycles of 95 °C for 15 s, and 60 °C for 1 min. Ct values of less than 36 were interpreted as positive [53].

To identify Brazilian alphaviruses and flaviviruses (MAYV, MADV and ROCV, SLEV, WNV, ILHV, YFV, BSQV, IGUV, respectively), we performed the duplex-nested PCR assay targeting a conserved domain of the nonstructural protein 1 (nsp1) and 5 (NS5) genes of Brazilian alphaviruses and flaviviruses, respectively [54]. The primers M2W-YAGAGCDTTTTCGCAYSTRGCHW and cM3W-ACATRAANKGNGTNGTRTCRAANCCDAYCC) anneal to the alphavirus nsp1 and primers FG1-TCAAGGAACTCCACACATGAGATGTACT and FG2-GTGTCCCATCCTGCTGTGTCATCAGCATACA anneal to the flavivirus NS5 gene. Precautions taken to avoid contamination have been described previously [55].

### 2.3. Serological Testing

Cerebrospinal fluid samples were screened for exposure to DENV, ZIKV, and CHIKV infection using the anti-Dengue IgM-ABCAM kit (Abcam plc, Cambridge, UK—ab108729), EUROIMMUN human anti-ZIKV IgM ELISA kit (EUROIMMUN, EURO-AG, Luebeck, Germany—EI 2668-9601 M), and EUROIMMUN human anti-CHIKV IgM kit (EUROIMMUN, EURO-AG, Luebeck, Germany—EI 293a-9601 M). All assays were performed according to the manufacturer’s recommendations using the corresponding positive and negative controls. ELISA plates were read using a Spectramax Plus ELISA reader at 450 nm (Molecular Devices, LLC, San Jose, CA, USA). Results were expressed in Standard Units (PU) and interpreted as <0.8 negative, >0.8 and <1.1 equivocal and >1.1 positive.

### 2.4. Ethical Approval

Ethics Committee approval was granted by the São José do Rio Preto School of Medicine (FAMERP), protocol number 81649317.5.0000.5415, approved on 24 May 2019.

## 3. Results

### 3.1. General Characteristics

We selected from the biobank of the Hospital de Base de São José do Rio Preto; 299 samples collected between January 2016 and December 2017 for further analysis. Based on the exclusion criteria defined above (Section 2.1) 44 samples were excluded, resulting in 255 samples for analysis, for which we have included demographic data (age and gender) and their clinical diagnoses. The age mean of the sample cohort was 30.2 years (±28.0) (range: 1 day old to 90 years), of which 60.4% (*n* = 154) were male. Meningitis was the most common clinical syndrome (*n* = 107, 42.0%) observed, followed by cerebrovascular events, recorded as bleeding or stroke (*n* = 42, 16.5%), and meningoencephalitis (*n* = 57, 22.4%); there was 1 case each of venous thrombosis (0.4%) and demyelinating polyneuropathy (0.4%).

### 3.2. Arboviral Infection Diagnosis

Through molecular and serological analyses, arboviruses were detected in 9 out of 255 samples, representing a rate of 3.53%. Of these, 8 (3.13%) were determined to be positive by RNA detection, 6 positive for ZIKV, and 2 for DENV. Neither CHIKV, ROCV, SLEV, WNV, MAYV, YFV, BSQV, MADV, nor IGUV were identified in the samples tested. During the study, a case of ILHV was identified and has already been described previously [9] and not included herein. Serological diagnosis was positive for 1/255 samples (0.39%), by the ZIKV IgM ELISA. Neither anti-dengue nor anti-CHIKV IgM antibodies were detected in any of the examined samples.

### 3.3. Clinical Findings

Table 1 summarizes the clinical findings of the nine arbovirus-positive patients. The age of the patients ranged from 18 to 74 years, seven of whom were adults [18–65 years old] and two of whom were elderly (older than 65). Of the positive cases, 55.5% (5/9) were women. Meningitis (3/9; 33.33%) and encephalitis (3/9; 33.33%) were the main clinical symptoms observed, followed by brain bleeding (2/9; 22.22%) and, in one case, demyelinating polyneuropathy (1/9; 11.11%).

Among the ZIKV-infected patients, three meningitis cases were identified, two of which were diagnosed by molecular testing (a ZIKV-positive PCR), and the third was identified by antibody detection (anti-ZIKV IgM positive). Three of these patients had underlying comorbidities, patients 253 and 257 were HIV positive, and patient 272 had hyper-IgD syndrome. In addition, one case of encephalitis (ID 194), two cases with brain bleeding (ID177 and ID 248), and one case of demyelinating polyneuropathy (ID 257) was associated with ZIKV (Table 1).

## 4. Discussion

Herein, we describe the incidence of arboviruses as a collective cause of neuroinvasive disorders in a region where they cocirculate and where DENV and ZIKV transmission is dominant. The study emerged from the need to identify such serious manifestations in a region of complex arbovirus transmission dynamics that is hyperendemic for all DENV serotypes [42,44,45,47], has had a previous ZIKV outbreak [51,56], CHIKV and SLEV [40,49] have been detected, and with high levels of YFV vaccination [57]. Such aspects have become crucial to the understanding of how arboviruses spread worldwide.

Globally, arboviruses with the potential for neuroinvasion cause neurological disease at a rate of 50/100,000 cases per year [58,59,60]. Studies have reported that 40% to 70% of patients maintained neuropsychological and functional impairments for years after an acute neurological episode [61,62,63,64]. In 50% of cases, the causative agent may not be identified [65,66]. In Brazil, some studies have found neurological disorders to be related to arboviral infections [5,67,68,69]; however, most of these studies do not describe a systematic surveillance system for detecting arboviruses and largely consist of case reports [70,71,72,73,74]. Encephalitis is the most common form of neuroinvasive disease [75], followed by polyradiculopathies [76]. The patients with arboviral infections detected in the present study most frequently exhibited symptoms of encephalitis and meningitis (each at a rate of 33.33%); these infections were also associated with two cases of brain bleeding (22.22%) and one case of demyelinating polyneuropathy (11.11%).

Often, suspected cases of viral meningoencephalitis are reported without diagnosis of its causative agent. The severity of such cases varies depending on the accuracy of the diagnosis, the etiological agent involved, and the age and immunological status of the patient [77]. The viruses most commonly associated with meningoencephalitis are herpes simplex, enteroviruses, and the varicella zoster virus, but there are also reports of arboviruses being associated with meningoencephalitis [16,77,78], as in the present study. In these cases, the most commonly reported clinical manifestations were meningitis and encephalitis, presentations that reinforce the relationship these conditions exhibit with DENV and ZIKV.

Between 2015 and 2019, the city of São José do Rio Preto reported 44,898 dengue cases [79] and 2169 Zika cases [80]. Although the Zika cases were of a lesser magnitude, the surveillance of arbovirus-related neurological findings detected Zika at a rate three times higher than that of dengue. This observation was noteworthy in itself because it suggested central nervous system involvement in ZIKV infection. The neurotropism and neurovirulence of ZIKV has been extensively documented since its 2015 re-emergence, mainly through cases of congenital Zika syndrome (CZS) [26,81]. These cases exemplify the serious impact of ZIKV infection on human fetal brain development. Furthermore, studies show that infection in children and adults is also linked to structural and functional neural abnormalities [82], such as the ability to infect and replicate in endothelial cells of the blood–brain barrier [83,84]. However, reports of cerebrovascular manifestations associated with Zika are rare and have been confined to case reports [33,70] and in the current study, in which we have diagnosed two cases.

It is challenging to determine clinical associations between arbovirus and neurological events and to establish etiological diagnoses. Herein, we identified a case (ID 248) in which the patient presented classic symptoms of acute ZIKV infection with a brain bleed, which corroborates other recent observations [70]. In contrast, we observed another case (ID 177) in which the patient exhibited no symptoms of arboviral infection, but the molecular RT-qPCR used in our study detected acute ZIKV infection with an ischemic vascular incident and in the absence of coagulation disorders. These cases demonstrate the complexity of establishing clinical correlations with arboviral infections and show that the etiological diagnosis can be even more difficult when the patient may not present symptoms of arboviral infection.

In addition to acute neurological involvement, post-infectious autoimmune disorders are also caused by arboviruses and are characterized by bilateral flaccid limb weakness attributable to peripheral nerve damage, which can lead to syndromes such as GBS and transverse myelitis [85,86]. Similarly, our study found a case (ID 257) of demyelinating polyneuropathy in which the patient was hospitalized due to ataxia and decreased lower limb strength. In this case, the electroneuromyography revealed an acute demyelinating pattern, and the CSF tested positive for ZIKV when RT-qPCR was used.

Three cases of ZIKV-positive patients were also HIV positive with AIDS syndrome, and one of these patients had leukopenia (2580 leukocytes/mm^3^). There are few reports in the literature on the relationship between ZIKV and HIV. A previous clinical case study described an autochthonous case of ZIKV infection in an HIV-infected patient in Brazil, in which the patient presented with mild symptoms [87]. However, there is also a case of ZIKV infection from the same region in which a ZIKV infection acquired during the first trimester in an HIV-infected pregnant woman led to multiple fetal malformations and fetal death [88]. Our study reflects the need for a greater understanding of this infection in different population groups and suggests that further studies should be performed in order to expand knowledge on arboviral infections in different populations.

Although the viruses cause a similarly broad range of CNS manifestations, our study shows that the use of laboratory tests may help health professionals to diagnose neuroinvasive diseases related to arboviruses such as DENV and ZIKV. Furthermore, the ILHV case found during our analyses showed that populations may be at risk for other underreported neuroinvasive arboviruses that may be circulating [9], since epidemiological surveillance does not typically cover all of the arboviruses considered herein (ROCV, SLEV, WNV, MAYV, ILHV, BSQV, IGUV and MADV) as potential causes of neurological manifestations.

This study exemplifies the usefulness of associating clinical and laboratory data to determine cases of arboviral infection associated with neuropathy. To achieve an accurate diagnosis, it is important to consider geography (the SJdRP region is hyperendemic for dengue [42,89] and multiple arboviruses cocirculate [40,89]), clinical data typical of arbovirus infection, and also laboratory data, such as CSF, cell and protein assessments, and information provided by molecular and serological assays.

The current study was carried out in a retrospective cross-sectional manner based on samples obtained from an institutional biobank. Our ability to detect arboviral genetic signatures in the CSF samples of patients with neurological manifestations suggests a strong correlation with arboviral infection albeit at low incidence rate. Prospective surveillance studies that include collection of serum and CSF samples in patients with neurologic manifestation will be critical, providing insights into the incidence and prevalence arbovirus infections contribute to neurological outcomes. Finally, we would like to reiterate to health care professionals the importance of establishing an accurate etiological diagnosis based on comprehensive laboratory analysis. Even if there is not yet a specific treatment for neuroinvasive disease caused by arboviruses, the true impact of these viruses on the population can become clearer with the acquisition of additional data, which in turn can guide future research. One reason for the absence of specific treatments may be the lack of understanding of the full spectrum of arboviral clinical presentations and specifically neurological presentations.

## 5. Conclusions

In conclusion, our study offers insights regarding the frequency of neurological manifestations following arbovirus infections. We have demonstrated a pathway based of commonly available laboratory tests to provide an accurate diagnosis and correlate neurological manifestations to arbovirus infections. Our retrospective study illustrates the importance of epidemiological surveillance of neuroinvasive arboviruses in accurately determining the true incidence and prevalence in hyperendemic regions, in order to guide public health officials and policy makers for improvements in public health planning and medical case management.

## Figures and Tables

**Table 1:** Clinical data from the positive patients in CSF samples.

ID	Gender, Age	Clinical Syndrome	Signs and Symptoms	CSF Characteristics	Other Observations	Diagnostic Test
144	Male, 61	Meningitis	Fever, myalgia, lethargy	Leukocytes 4 cells/mm^3^, lymphocytes 88%, neutrophils 2%, protein 44 mg/dL, glucose 90 mg/dL, culture negative	Leukopenia (2580 leukocytes/mm^3^); comorbidity: HIV infection	ZIKV_Elisa_ IgM positive
177	Female, 71	Brain bleeding	Headache	Leukocytes 11 cells/mm^3^, lymphocytes 67%, neutrophils 10%, protein 90 mg/dL, glucose 64 mg/dL, culture negative	No coagulation disorders detected	ZIKV_PCR_ positive
194	Male, 74	Encephalitis	Lethargy, mental confusion and decreased consciousness awareness	Leukocytes 17 cells/mm^3^, lymphocytes 67%, neutrophils 23%, protein 161 mg/dL, glucose 21 mg/dL, culture negative		ZIKV_PCR_ positive
248	Female, 59	Brain bleeding	Fever, náusea, irritability, respiratory distress	Leukocytes 89 cells/mm^3^, lymphocytes 74%, neutrophils 6%, protein 71 mg/dL, glucose 82 mg/dL, culture negative		ZIKV_PCR_ positive
253	Male, 56	Meningitis	Fever, lethargy, dyspnea, mental confusion	Leukocytes 39 cells/mm^3^, lymphocytes 50%, neutrophils 38%, protein 98 mg/dL, glucose 41 mg/dL, culture negative	Comorbidity: HIV infection	ZIKV_PCR_ positive
257	Female, 51	Demyelinating polyneurophaty	Myalgia, peripheral facial palsy, ataxy, decreased lower limbs strength	Leukocytes 11 cells/mm^3^, lymphocytes 95%, neutrophils 5%, protein 187 mg/dL, glucose 47 mg/dL, culture negative	Comorbidity: HIV infection	ZIKV_PCR_ positive
272	Male, 18	Meningitis	Fever, headache	Leukocytes 29 cells/mm^3^, lymphocytes 76%, neutrophils 6%, protein 81 mg/dL, glucose 79 mg/dL, culture negative	Comorbidity: Hyper IgD syndrome	ZIKV_PCR_ positive
04	Female, 50	Encephalitis	Aphasia, mental confusion, convulsive crisis and decreased consciousness awareness	Leukocytes 2 cells/mm^3^, lymphocytes 80%, neutrophils 8%, protein 91 mg/dL, glucose 69 mg/dL, culture negative		DENV_PCR_ positive
06	Female, 65	Encephalitis	Speech difficulty, drowsiness, decreased lower limbs strength	Leukocytes 1 cells/mm^3^, lymphocytes 85%, neutrophils 5%, protein 44 mg/dL, glucose 251 mg/dL, culture negative		DENV_PCR_ positive

CSF: cerebrospinal fluid; reference values: Leukocytes ≤ 3 cells/mm^3^, lymphocytes ≥ 90%, neutrophils ≤ 10%, protein ≤ 40 mg/dL, glucose ≥ 2/3 serum level, culture negative.

## Data Availability

De-identified data are available from the authors upon request.
